# Inadequate management of pneumonia among children in South Ethiopia: findings from descriptive study

**DOI:** 10.1186/s12913-019-4242-7

**Published:** 2019-06-26

**Authors:** Solomon Hailemariam, Yabibal Gebeyehu, Eskindir Loha, Kjell Arne Johansson, Bernt Lindtjørn

**Affiliations:** 1 School of Public Health, Dilla University, Dilla, Ethiopia; 2 Paediatrics Department, School of Medicine, Dilla University, Dilla, Ethiopia; 3 School of Public Health, Hawassa University, Hawassa, Ethiopia; 4 Department of Global Public Health and Primary Care, University of Bergen, Bergen, Norway

**Keywords:** Health system, Managing pneumonia, Children, Ethiopia

## Abstract

**Background:**

Health system support is crucial for quality child healthcare. Therefore, this baseline survey, which is part of the community-based management study of severe pneumonia, was conducted to assess the state of health system support of IMNCI and iCCM, and health workers’ knowledge in managing childhood pneumonia at health facilities.

**Methods:**

A survey was conducted in 99 government health institutions in South Ethiopia from 07 to 14 January, 2018. A questionnaire for health system support and case scenario for the management of severe pneumonia was adapted from the WHO health facility survey tool. The questionnaire’s interview, facility observation, case scenario and retrospective record review were all used as data collection methods. Indicators of health system support in the context of an integrated management of childhood illness were used. Proportions for categorical variables and means for continuous variables were also computed for each indicator. Mean score was analysed for assessing the knowledge of health workers in managing the case scenario.

**Results:**

In the study area, only 12 (34%) of health centres and 18 (29%) of health posts received supervision, which included the observation of case management. The mean number of essential oral antibiotics for the home treatment of pneumonia available at the facility was 1.1 (95% CI 0.9 to 1.3), whereas the mean number of pre-referral drugs for the treatment of severe pneumonia was 1.3 (95% CI 1.0 to 1.6). Approximately 47 (48%; 95% CI 37.7 to 57.3) of the surveyed health facilities had materials and equipment to support vaccination services, and 71 (72%; 95% CI 62.8 to 80.6) of them had the vaccines on the day of the survey. Only four (4%; 95% CI 0.3 to 8.3) of the health facilities had all the essential job aids and supplies for providing services for pneumonia. The providers’ mean knowledge score for the management of severe childhood pneumonia was 14.9 out of 22 correct answers.

**Conclusion:**

There is a room to improve the health system support to integrated management of neonatal and childhood illness through supply chain management and knowledge of health workers in the management of severe pneumonia by providing training.

## Background

Pneumonia remains one of the leading causes of death in children under 5 years of age [1]. In 2016, 5.6 million children aged less than 5 years died, with pneumonia accounting for 16% of all global deaths [2].

To reduce child mortality in low-income countries, the World Health Organization (WHO) and the United Nations International Children’s Emergency Fund (UNICEF) developed a strategy known as the integrated management of childhood illness (IMCI) in the mid-1990s, [3]. In 2003, care for newborns under one week of age was added, with the strategy known as the integrated management of neonatal and childhood illness (IMNCI) [4]. The IMNCI strategy has three components: 1) improving the skills of health workers in case management; 2) strengthening the health system; and 3) promoting family and community health practice through recommended actions at the household and community levels [5].

The first component combines curative interventions for common childhood illnesses such as pneumonia, diarrhoea, malaria, malnutrition, measles, anaemia, meningitis, sepsis and ear infection, as well as preventive interventions such as immunization, nutrition counselling and breastfeeding support [6]. The second component puts an emphasis on essential elements of the health systems that ensure effective and quality child curative intervention [5]. It ensures an availability of drugs, IMNCI planning and management, the availability of essential equipments and materials, vaccines, supervision and health workers trained in IMNCI [5, 7]. The third component of IMNCI is an approach intended to improve community and household health practice [1].

Studies have shown that the quality of management of common childhood illnesses has improved after the implementation of WHO IMNCI guidelines [8, 9], as mortality in children younger than 5 years of age has decreased by 15% [10]. However, IMNCI training alone is not sufficient to provide and maintain quality child healthcare [6]. Despite the wide IMNCI training coverage, the quality of care has been remained low [11]. Improving and sustaining quality child healthcare needs the convergence of a number of factors together [1]. IMNCI training, with supports like supervision [5, 11, 12], the availability of recommended drugs [1, 5, 13], the availability of equipment [1, 14] and the availability of counselling guide [11], are important factors for providing quality child healthcare at first-level health facilities.

Ethiopia has reduced mortality among children less than 5 years of age by 71% over the last few decades (from 191 deaths per 1000 live births in 1990 to 55 deaths per 1000 live births in 2015) [15]. There are 15 deaths per 1000 live births due to pneumonia in Ethiopia, and the country ranks 6th among the top 15 countries in morbidity and mortality from pneumonia globally [16].

In Ethiopia primary hospital, health centres, and health posts make up the primary health care unit [17]. Health post is staffed by two female health extension workers from the nearby villages, having completed at least 10th grade. They receive a 12 month theoretical and practical training on 16 packages to provide preventive, promotive, and curative activities on selected maternal and child health [18]. A health centre is a facility at primary level of the health care system which provides promotive, preventive, curative and rehabilitative outpatient care for population including basic laboratory and pharmacy services with the capacity of 10 beds for emergency and delivery services. As a strategy to reduce under 5 years of age child mortality, Ethiopia implemented the integrated management of neonatal and childhood illness (IMNCI) at health centres and hospitals [17]. Since 2010 Ethiopia has also implemented integrated community case management (iCCM) at health posts by health extension workers [19], to provide curative interventions to sick children by health extension workers at health posts [18]. Refer severe children to health centres and health workers at health centres supervise and support health extension workers at health posts [20].

In Ethiopia, the health system support of integrated community case management (iCCM) was evaluated in health posts, with findings from these studies showing that the status of the health system was not sufficient to provide quality child healthcare [12, 18, 21]. However, these studies lack evidence about the health system support of IMNCI from health centres, and there is limited information on the management of acute respiratory tract infections. It is not known whether the health system support of IMNCI strategy is being implemented successfully or not. The findings from this survey identified gaps for possible intervention to strengthen the IMNCI/iCCM strategies. Therefore, this survey, which is part of a cluster randomized controlled trial for improving the management of severe pneumonia, aims to assess the state of health system support of IMNCI and iCCM, and health workers’ knowledge in managing childhood pneumonia at health facilities.

## Methods

### Study design

A descriptive study design was used.

### Setting

**Fig. 1:**
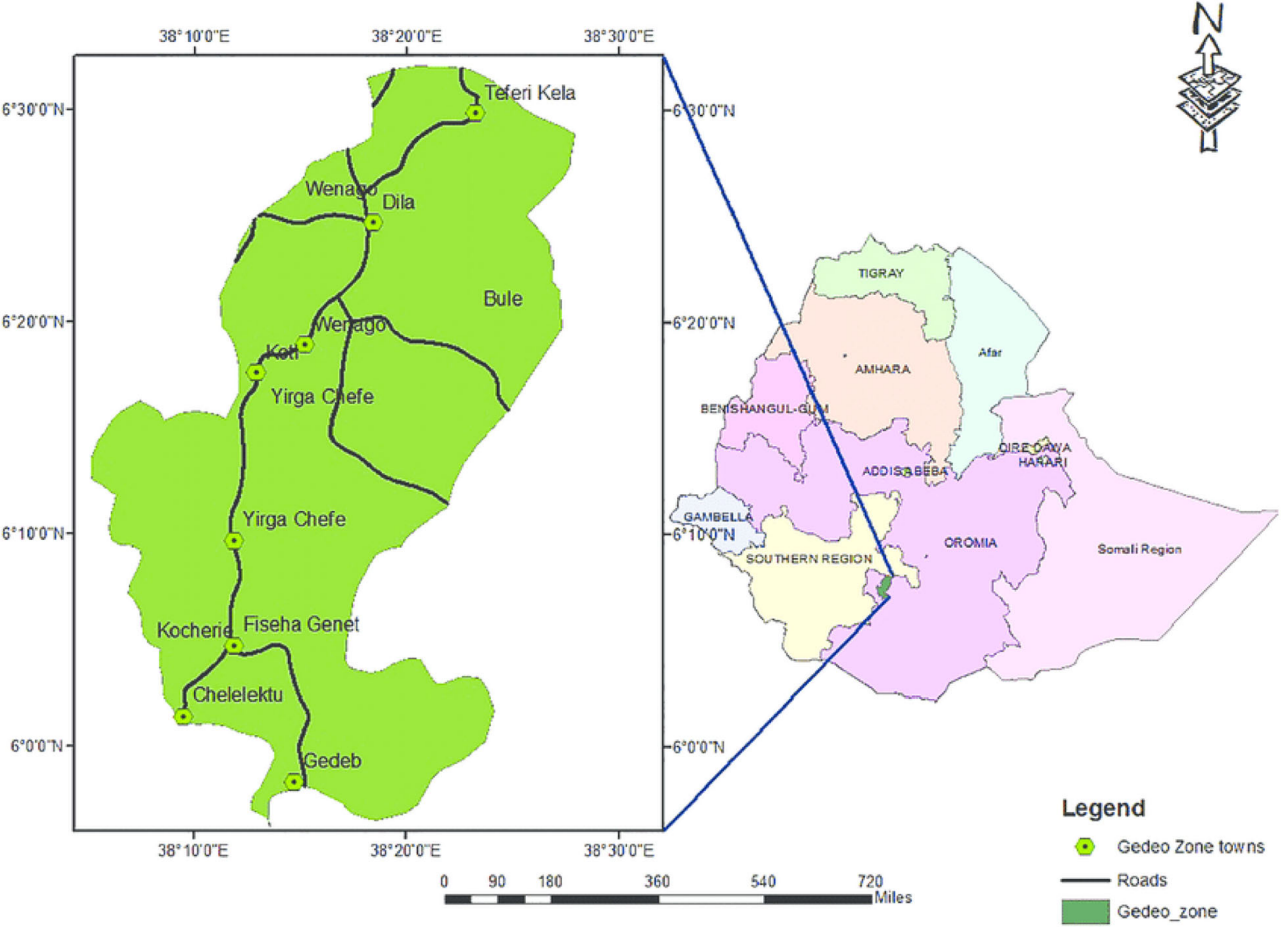
Map of study area, South Ethiopia and Gedeo zone, with permission from Gedeo Zone administrative office

The Federal Democratic Republic of Ethiopia is composed of nine Regional States and two City Administrations council. The regional states and city administrations are subdivided into Zones and this further subdivided into administrative Woredas (districts). A Woreda/District is the basic decentralized administrative unit. The Woredas are further divided into about Kebeles, the smallest administrative unit in the governance [17]. We collected data on the health system support components of IMNCI/iCCM through a cross-sectional survey of rural health posts, health centres and a hospital in the Gedeo Zone in South Ethiopia (Fig. 1). The area is located 360 km from Addis Ababa. The Zone has an area of 1210.89 km^2^. The total population of the zone is 1.1 million people, and of these 173,700 are children under 5 years of age. The zone is composed of 7 Woredas. Cash crop like coffee is the main source of income [22]. In the area there are 146 health posts, 38 health centres, and 1 hospital. All health centres are using the WHO-IMNCI guidelines to manage pneumonia, and iCCM is implemented at health posts. The survey was conducted from 7 to 14 January, 2018.

### Participants

According to the WHO health facility survey tools [23], we sampled 101 health facilities (35 health centres, 65 health posts, and one hospital). A stratified sample of health facilities was selected for facility level data. Probability proportional to size sampling was used to randomly select health centres, health posts and hospital, while the random selections of facilities were carried out using “Random.Org”. To be eligible, health facilities had to be found within 15 km from the district health office. Accordingly, for facility level variables from 146 health posts implementing iCCM, 96 of the facilities were eligible for random selection, and 65 health posts were selected. Similarly, from 38 eligible health centres implementing IMNCI, 35 health centres were selected. The only available referral hospital (Dilla Hospital) was selected. From selected health facilities, health workers were also included in the study for case scenario. To be eligible health workers, they had either been trained in IMNCI or were working in outpatient departments for children on the day of the survey.

### Variables and data collection methods

The indicators of IMNCI for health service system support and case scenarios for the management of severe childhood pneumonia were adapted from the WHO health facility survey tool [23], and key indicators of iCCM health system support were adapted from global indicators [24]. Table 1 shows the list of indicators and their definitions, with measurement methods and sources for each variable. We added some equipment like oxygen cylinder, X-ray machine, and pulse oximetry during data collection, as these materials are important for pneumonia management. But these equipments were not used during analysis for indicators. Since our study focus on management of pneumonia, we omitted some indicators used for other childhood illnesses.

**Table 1:** Definition of indicators

Indicators	Metric	Definitions	Measurement method/sources	Categories measured
Supervision	Numerator: Number of health facilities that received at least one visit of routine supervision (excluding the follow-up visits to health workers shortly after their training that are part of IMNCI training), which included the observation of case management during the previous three/six months Denominator: Number of health facilities surveyed.	Proportion of facilities received at least one supervisory visit that included the observation of case management during the previous six months for IMNCI trained staffs, and three months for iCCM trained health extension workers	Health workers questionnaire interview	
Essential oral treatment for pneumonia	Numerator: Number of first-line oral treatments available the day of visit Denominator: 2	Arithmetic mean of essential oral drugs recommended for the home treatment of pneumonia available at each facility the day of visit	Direct observation Stocks	• Amoxicillin syrup/suspension • Cotrimoxazole syrup/suspension
Injectable drugs for pre-referral treatment of pneumonia	Numerator: Number of injectable drugs for pre-referral treatment available the day of visit Denominator: 4	Arithmetic mean of recommended injectable pre-referral treatment for children and young infants with severe classification needing immediate referral divided by four for IMNCI and divided by one for iCCM.	Direct observation Stocks	• Ampicillin • Chloramphenicol • Gentamycin^*^ • Benzylpeniciline
Equipment and supplies for vaccine	Numerator: Number of health facilities that have the equipment and supplies to support full vaccination services on the day of survey Denominator: Number of health facilities surveyed	Proportion of health facilities that have the equipment and supplies to provide full vaccination services on the day of survey	Direct observation Vaccination room and stocks	• Functioning refrigerator or cold chain • Needles/syringes
Key vaccines	Numerator: Number of the recommended vaccines available the day of visit Denominator: 5	Arithmetic mean of recommended vaccines available at each facility the days of visits	Direct observation Vaccination room and stocks	• BCG • Polio • DPT-HepB-Hib • PCV • Measles
Trained IMNCI	Numerator: Number of health facilities with at least 60% of health workers managing children who are trained in IMNCI Denominator: Number of health facilities surveyed	Proportion of health facilities with at least 60% of health workers managing children trained in IMNCI	Head of facility questionnaire interview	
iCCM trained health extension workers (HEW) density	Numerator: Number of HEWs who are trained and deployed (to serve in a specific target area) Denominator: Number of children under five in target communities **÷** 1000	Number of CHWs trained and deployed for iCCM per 1000 children under 5 years of age in target areas	Head of facility questionnaire interview	
Correct case management for severe pneumonia (knowledge)	Numerator: Number of health workers who respond to correct management for case scenario Denominator: Number of health workers assessed	Proportion of health workers who gave correct response for the management of case scenario	Application of case scenario to health workers	
All essential job aids and supplies for the management of pneumonia	Numerator: Number of health facilities with all the needed equipment and materials Denominator: Number of health facilities surveyed	The proportion of health facilities that have all the needed equipment and materials available on the day of the survey.	Direct observation of outpatient department	• Respiratory timer • Thermometer • Syringe and needles • IMNCI/iCCM chart booklet • Mother’s counselling cards
Supervisory visits received		Mean (and range) of the number of supervisory visits received for IMNCI in the last six months and for iCCM in the last three months	Health worker questionnaire interview	
Facility open		Mean (and range) for: days per week the facility is open	Health worker questionnaire interview	
Child services		Mean (and range) for: days per week child health services are provided	Health worker questionnaire interview	
Vaccination services		Mean (and range) for: days per week vaccination services are available	Health worker questionnaire interview	
Time travel to nearest referral facility		Mean (and range) of the time required to get to the nearest referral facility	Health worker questionnaire interview	
Case load		Median (and range) of the number of sick child visits during the previous month	Record review	
IMNCI trained	Numerator: the total number of staff managing children trained in IMNCI Denominator: the total number of staff assigned to the case management of children.	The proportion of staff trained in IMNCI actually present and managing children the day of visit	Head of facility questionnaire interview	

* for iCCM at Health post mean and range was computed based on only Gentamycin

For the case scenario, we used correct case management (knowledge) as an indicator. Twenty-two questions on the management of severe childhood pneumonia were asked. The case scenario describes the child who present to the health facility. This provides some information about the health worker knowledge of case management for severe pneumonia. All the actions that are needed to provide appropriate treatment for severe pneumonia were included. Whether to refer the case or not, action need to be taken along the referral, drugs and other management was included. A health worker is correctly identifying treatment for severe pneumonia if: 1) he or she identified all recommended treatments and no unnecessary treatments; and 2) referred the case to a hospital.

The data collection methods include questionnaire interview, facility observation, case scenario and retrospective record review. Data collectors reviewed the integrated management of neonatal and childhood illness records. The total number visits, and visits for pneumonia at health facilities in the previous month, were recorded from the registration book. Trained data collectors who completed 12th grade did the data collection.

### Data analysis

Data was doubled entered into Epi data version 3.1 software, and all analyses was carried out in SPSS version 20 (IBM Corp., Armonk, N.Y., USA). Each indicator analysis was computed according to WHO health survey facility tool [23], and for categorical variables proportions with a 95% confidence interval were used. For continuous variables, means and range with 95% confidence intervals were used. To account for the probability proportional to size allocation, we calculated both weighted and unweighted overall results.

The mean score was analysed for assessing the knowledge of health workers in managing the case scenario. In addition, to see the predictors of knowledge, we constructed a linear mixed level regression. Individual level variables such as age, sex and IMNCI training, as well as facility level variables such as number of supervision visits was included in the model. Moreover, IMNCI training variable was considered as random variable at facility level.

## Results

### Study response

Facility-based data was available from 99 health facilities (one referral hospital, 35 health centres and 63 health posts), achieving a total facility response rate of 98%. At one health post, the key informant was not available and one health post was not functioning. From the selected health facilities, 125 of the 162 (77%) health workers, who were either IMNCI trained or managed children, were interviewed for the case scenario (Table 2).

**Table 2:** Study sample and response rate, Gedeo Zone, South Ethiopia, 2018

Facilities visited						
Facility type	Total number		Sample	Response rate *n* (%)		
Hospital	1		1	1 (100)		
Health centre	38		35	35 (100)		
Health post	146		65	63 (97)		
Total	185		101	99 (98)		
Case scenario						
Facility type	Number of doctors	Number of health officers	Number of nurses	Number of health extension workers	Number trained in IMCI and/or in managing children	Response rate *n* (%)
Hospital	46	6	104	0	12	2 (17)
Health centre	10	101	379	0	70	54 (77)
Health post	0	0	0	102	80	69 86)
Total	56	107	483	102	162	125 (77)

### IMNCI/iCCM indicators

Table 3 shows indicators for IMNCI/iCCM at facility levels, with the results for each of the indicators as follows:

**Table 3:** Facility-specific and overall summary results of IMNCI/iCCM indicators, Gedeo Zone, South Ethiopia, 2018

Indicators	Hospital *n* (%)	Health centres *n* (%)	Health posts *n* (%)	Unweighted % (95% CI)	Weighted % (95% CI)
Supervisory visit that includes observation of case management	0 (0)	12 (34)	18 (29)	NA^*^	NA^*^
Four pre-referral injectable drugs	1 (100)	13 (37)	NA^*^	14 (7–21)	40 (30–39)
Equipment and supplies to provide full vaccination services	1 (100)	35 (100)	11 (18)	48 (38–57)	35 (25–44)
Vaccines available the day of visit	1 (100)	35 (100)	35 (56)	72 (63–81)	65 (56–74)
Facilities with at least 60% staff managing children trained in IMNCI	0 (0)	21 (60)	NA^*^	58 (49–67)	58 (48–68)
Staff trained in IMNCI/iCCM actually present and managing children the day of visit	0/205 (0)	67/600 (11)	79/102 (77)	16 (14–18)	23 (20–26)
iCCM health extension workers density	NA^*^	NA^*^	1.2 per 1000 children	–	–
Essential job aids and supplies for the management of pneumonia	0 (0)	1 (3)	3 (5)	4 (0–8)	4 (0–8)
	Mean (range)	Mean (range)	Mean (range)	Mean (95% CI), range	Mean (95% CI), range
Essential oral treatments for the treatment of pneumonia	2.0 (2–2)^**^	1.8 (0–2)	0.6 (0–2)	1.1 (0.9, 1.3), 0–2	1.2 (0.6, 1.7), 0–2
Injectable drugs pre-referral treatment	4.0 (4–4)^**^	2.8 (1–4)	0.4 (0–1)	1.3 (1.0, 1.6), 0–4	1.8 (1.1, 2.5), 1–2
Number of key vaccines availability	5.0 (5–5)^**^	5.0 (5–5)	3.0 (0–5)	3.7 (3.3, 4.1), 0–5	3.9 (2.6, 5.2), 2–5
Number of vaccines for the prevention of pneumonia available	2.0 (2–2)^**^	2.0 (2–2)	1.2 (0–2)	1.5 (1.3, 1.7), 0–2	1.6 (1.1, 2.1), 0–2
Number of days per week the facility is open	7 (7–7)^**^	7 (7–7)	5.1 (3–7)	5.8 (5.6, 6.0), 3–7	6 (5.5, 6.5), 4–7
Number of days per week child health services are provided	7 (7–7)^**^	6.8 (5–7)	5.0 (3–7)	5.7 (5.5, 5.9), 2–7	5.9 (5.1, 6.7), 4–7
Number of days per week vaccination services are available	5 (5–5)^**^	1.8 (1–3)	1.2 (1–3)	1.4 (1.3, 1.5), 1–5	2.2 (1.8, 2.6), 1–3
Number of supervisory visits received in the last six months.	1 (1–1)^**^	3.2 (0–12)	2.9 (0–12)	3 (2.4, 3.6), 0–12	2.5 (− 0.4, 5.4), 0–9
Number of supervisory visits received in the last six months for IMNCI-trained health workers, and in the last three months for iCCM-trained health extension workers	0 (0)	1.0 (0–4)	0.8 (0–12)	0.9 (0.5, 1.3), 0–12	0.7 (− 0.9, 2.3), 0–7
Hours required to get to the nearest referral facility	NA^*^	1.9 (0–12)	1.6 (1–15)	1.7 (1.2, 2.2), 0–15	1.7 (− 0.6, 3.9), 0.6–14
Number of sick child consultations in the previous month	308 (308–308)^**^	65 (10–323)	19 (3–78)	38 (28, 48), 3–323	97 (69, 125), 75–192

*Not applicable

** number of hospital is one

#### Supervision of health facilities

The referral hospital did not receive any supervision in the last six months prior to the survey, whereas 12 (34%) of the health centres and 18 (29%) of the health posts did receive at least one supervisory visit that included the observation of case management during the previous six months for health centres and three months for health posts.

#### Essential medicines for the treatment of childhood pneumonia

The surveyed hospital had all the recommended oral and parenteral antibiotics for the treatment of pneumonia. The mean (range) number of available essential oral drugs (Amoxicillin, and co-trimoxazole) for the home treatment of children with pneumonia at the facility on the day of visit was 1.8 (0–2) for health centres, while for health posts this figure was 0.6 (0–2) out of the total mean score of 2. Similarly, the mean (range) number for the availability of four parenteral antibiotics drugs (Ampicillin, Gentamycin, Chloramphenicol and Benzyl penicillin) for the pre-referral treatment of severe pneumonia on the day of the visit was 2.8 (1–4) for health centres out of a total score of 4 and 0.4 (0–1) for health posts out of a total score of 1. Overall, only 14 (14%) of surveyed health facilities had all four parenteral antibiotics available on the day of the visit.

#### Vaccines and supplies

All the visited health centres and the hospital had all the necessary supplies to fully support the vaccination programme, while only 11 (18%) of the visited health posts had the equipment and material for the vaccination programme. Except for the health posts, both the surveyed hospital and health centres had all the vaccines on the day of visit, but the mean index of availability of the vaccines in the visited health posts was 3.0 (range 0–5) out of a total score of 5. Moreover, the mean availability of the two important vaccines (Haemophilus influenza type b and pneumococcal conjugate vaccine) for the prevention of pneumonia was 1.2 (range 0–2) out of a total score of 2. Overall, approximately two-thirds of the surveyed health facilities in the study area had all five vaccines.

#### Essential job aids and supplies

Among the four essential job aids and supplies of IMNCI/iCCM important for providing service for pneumonia management, only four (4%) of the facilities had the job aids and supplies for the programme.

#### Service availability

The hospital and all visited health centres were open the entire week, but the mean days per week that the health posts were open was 5.1 (range 3–7). Child health services were not provided for the entire week in health facilities, except in the hospital. The mean days per week for providing vaccination services were 1.8 (range 1–3) days in health centres and 1.2 (range 1–3) days in health posts. The mean time required to get to the nearest referral facility was 1.9 (range 0–12) hours for health centres and 1.6 (range 0–15) hours for health posts.

#### IMNCI/iCCM training coverage

From 907 health workers deployed at the health facilities, only 146 (16%) of the staff trained in IMNCI/iCCM were actually present and managing children on the day of visit. From the total health centres surveyed, 60% of the facilities had at least 60% of their health workers managing children trained in IMNCI. There were 1.2 health extension workers trained and deployed for iCCM per 1000 children under the age of 5 years.

#### Caseload

The total number of children who made visit in 99 health facilities at the sick child clinic was 3744. Of these, 3464 children were 0 to 5 years of age. The mean number of sick child visits per facility during the previous month was 35 (range 3–323). Of the total visits, 3205 (93%) visits were made by children from the age of two to 59 months. Of these, 1585 (49%) visits were made by girls. Out of 3205 visits, 1390 (43%) visits were for cough or difficult breathing, with the mean number of sick child per facility in the previous month being 14 (range 0–147) visits. Out of these, 66 (5%) visits were severe pneumonia, 948 (68%) mild to moderate pneumonia and 376 (27%) a common cold. Only 18 (27%) of the children with severe pneumonia were referred to the next higher referral institution.

In addition to IMNCI/iCCM indicators, we computed the proportion of health facilities that had each of the pieces of equipment and material, vaccines, supplies and essential medications available on the day of the survey.

### Key equipment and materials

The hospital had no equipment and materials such as a working watch for health workers managing children, mothers’ counselling cards and the IMNCI chart booklet, although the mothers’ counselling cards and IMNCI chart booklet were available at the majority of the visited health centres and health posts. Only one health centre had a pulse oximeter, but none of the health posts had this material. Oxygen cylinders or concentrators were only available in the hospital, whereas needles and syringes were only available in 48 (76%) of the health posts (Table 4).

**Table 4:** Percentage of health facilities that had each of the key equipment and materials, Gedeo Zone, South Ethiopia, 2018

Items	Hospital (1)	Health centre (35)	Health post (63)	Total (99)
Working watch/timing device	0	1 (3)	15 (24)	16 (16)
Mothers’ counselling cards	0	25 (71)	50 (79)	75 (76)
IMCI chart booklet	0	34 (97)	62 (98)	96 (97)
Accessible mean of transportation for referral patients	1 (100)	12 (34)	0 (0)	27 (27)
Needles and syringes for pre-referral drugs	1 (100)	35 (100)	48 (76)	84 (85)
Pulse oximetry	1 (100)	1 (3)	0 (0)	2 (2)
Oxygen cylinder/concentrator	1 (100)	3 (9)	0 (0)	4 (4)
Thermometer	1 (100)	35 (100)	57 (91)	93 (94)
Chest x-ray machine	1 (100)	0 (0)	NA	1 (1)
Number beds for children	44	53	NA	97

### Key vaccines and vaccination supplies

Vaccines, a functioning refrigerator and needles and syringes for vaccinations were available at all health centres and the hospital, although functioning refrigerators were only available in 11 (18%) of the health posts (Table 5).

**Table 5:** Percentage of health facilities that had each of the key vaccines and vaccination supplies in stock on the day of the survey, Gedeo Zone, South Ethiopia, 2018

Items	Hospital (1)	Health centre (35)	Health post (63)	Total (99)
Vaccination card	1 (100)	31 (87)	49 (78)	81 (82)
Needle and syringe	1 (100)	35 (100)	57 (91)	93 (94)
Functioning fridge	1 (100)	35 (100)	11 (18)	47 (48)
BCG	1 (100)	35 (100)	35 (55)	71 (72)
OPV	1 (100)	35 (100)	39 (62)	75 (76)
DPT-HepB-Hib	1 (100)	35 (100)	39 (62)	75 (76)
PCV	1 (100)	35 (100)	39 (62)	75 (76)
Measles	1 (100)	35 (100)	39 (62)	75 (76)

Table 5: Percentage of health facilities that had each of the key vaccines and vaccination supplies in stock on the day of the survey, Gedeo Zone, South Ethiopia, 2018.

### Essential medications available on the day of the survey

Essential oral and parenteral antibiotics were available at the hospital, and each of these drugs was available in most of the health centres (Table 6). The antibiotics recommended for the treatment of childhood pneumonia were available in less than half of the health posts.

**Table 6:** Percentage of health facilities that had each of the essential medications available on the day of the survey, Gedeo Zone, South Ethiopia, 2018

Drugs	Hospital (1)	Health centre (35)	Health post (63)	Total (99)
Oral Amoxicillin	1 (100)	33 (94)	27 (43)	61 (62)
Oral co-trimoxazole	1 (100)	30 (86)	12 (19)	43 (43)
Parenteral Ampicillin	1 (100)	19 (54)	NA	22 (22)
Gentamycin	1 (100)	33 (94)	23 (37)	57 (58)
Parenteral chloramphenicol	1 (100)	26 (74)	NA	27 (27)
Benzyl penicillin	1 (100)	21 (60)	NA	22 (22)
Sterile water for injection	1 (100)	34 (97)	5 (8)	40 (40)

### Case scenario

#### Characteristics of health workers

Of the 125 health workers interviewed, one was a medical doctor, 13 were health officers, 42 were nurses and 69 were health extension workers. Their mean (SD) age was 27 (4.6) years. They had a median of five years (range: 1–15 years) of working experience in managing childhood illnesses. Of the 125 interviewed providers, 103 (82%) had IMNCI/iCCM training. The median number of years since having received IMNCI training was four years (range: 1–11 years).

#### Knowledge in the management of severe pneumonia

Of the 125 health workers, 95 (76%) correctly classified the case scenario. The mean knowledge score in the management of severe childhood pneumonia was 14.9 out of 22 correct answers. Among the 95 health workers who correctly classified the case scenario, 94 (99%) knew that the case needed an urgent referral, but only 58 (61%) of the health workers identified the correct treatment for the case scenario. Looking at the knowledge in the management of severe pneumonia; 51 (74%) of health extension workers correctly classified the case scenario, and 20 (39%) correctly identified the recommended treatment. Forty-four (79%) health workers correctly classified the case scenario, 38 (86%) correctly identified the treatment of severe pneumonia according to the IMNCI algorithm.

##### Predictors of knowledge score at the individual and facility level

The constructed linear mixed level regression with fixed and random effect models showed that gender and the number of supervision visits were significant predictors of the knowledge score for the management of severe childhood pneumonia. Age, IMNCI training and supervisor observing case management were not statistically significant (Table 7). The residual variance was 2.16, much larger than the variances for the random intercept (1.79) and random slope (0.19), thereby indicating more variation within health facilities than between facilities. Controlling for the effect of facilities and health workers level data, nearly 8% of the variation in knowledge score was between rather than within the health facilities.

**Table 7:** Predictors of the knowledge score of health workers for the management of severe childhood pneumonia: Linear mixed level model, Gedeo Zone, South Ethiopia, 2018

Variable	Coefficient	s.e.	z	*P*	95% CI
Gender: female	−1.2	0.452	−2.65	0.008	−2.08 to −0.31
Age (years)	−0.07	0.05	−1.46	0.143	−0.16 to 0.02
IMCI training: yes	−0.67	0.52	−1.3	0.193	−1.68 to 0.34
Number of supervision	0.4	0.17	2.32	0.02	0.06 to 0.73
Supervisor observing case management: yes	0.54	0.54	1	0.316	−0.52 to 1.59
Constant	17.64	1.4	12.57	0	14.89 to 20.39
Random slope	0.19	0.46	–	–	0.00 to 23.54
Random intercept	1.79	1.33	–	–	0.42 to 7.89
Residual variance	2.16	0.59	–	–	1.27 to 3.63

## Discussion

Our study suggests that the current status of health facilities in the provision of services for the management of childhood pneumonia is poor.

One of the strengths of this study is verifying the availability of supplies and drugs through direct observation. Second, we used globally standardized indicators of the programme. Third, unlike previous studies, this study includes both primary and secondary health facilities, which will give a comprehensive picture of the situation of the health system support for the management of pneumonia in children.

The study is not without its limitations. Firstly, excluding health posts that are difficult to access might have introduced selection bias. Nevertheless, 96 (65%) out of the 146 available health post were included for random selection. We also believe health workers’ characteristics (professionals deployed at each health facility, years of training and IMNCI training duration), and the availability of supplies, materials and drugs are similar in other health facilities. Therefore, the selection bias in this study is low. Secondly, the gold standard approaches used to assess the knowledge of health workers in managing childhood illness is direct observation of consultation with clinical re-examination [23]. However, this approach requires substantial resources and may not be feasible. Hence, the current knowledge of health workers in managing severe pneumonia using the case scenario might not reflect their true knowledge. Other alternative approaches like the case scenario can also be used. Lastly, the availability of drugs, supplies and materials was assessed for the day of the visit; consequently, the result might indicate the status of the institution for that particular day.

Supportive supervision is thought to have a positive effect on health workers’ performance [25]. Thirty percent of the health posts received at least one supervision visit, but the standard is that > 90% of the health posts should receive at least one routine supervision visit every quarter, and that action is needed if it is < 75% [24]. The proportion of health posts which received supervision that includes the observation of case management was low compared to previous studies conducted in Ethiopia. Two studies found that 75% [18], and 85% [26] of health posts received supervision. The reasons for this difference is due to the fact that data was collected through the reviewing of reports of two years, while the other study used health professionals who had worked as iCCM trainers and supervisors for the health workers implementing iCCM. As a result, they might tend to create a more positive scenario than what is true in the institutions. Still, the proportion of health centres that received supervision in the last six months was almost similar to a recent survey [25] from 94 countries. This study indicated that less than 25% of the facilities had at least one supervision during the previous six months.

A direct comparison of the findings for the availability of pre-referral drugs with previous findings might be difficult because of the difference in the categories of drugs measured and the types of facilities where the studies were conducted. We found a shortage of the oral amoxicillin drug in health posts, as well as pre-referral drugs in both health posts and health centres. Oral amoxicillin is the first drug of choice for the treatment of childhood pneumonia in Ethiopia [27]. The World Health Organization recommends parenteral ampicillin or penicillin plus gentamycin for the treatment of severe pneumonia [28], with the standard treatment guideline in Ethiopia recommending benzyl penicillin or chloramphenicol for the treatment of severe childhood pneumonia at the health centre level [27]. In Ethiopia, health posts are expected to have parenteral gentamycin as the pre-referral drug, but only 37% of the health posts had it. In Ethiopia, stock outs are common in health posts [18].

The referral of children with severe pneumonia should be facilitated by administering pre-referral drugs [18]. But in this particular study area, in addition to a significance absence of pre-referral drugs in the facilities, the time for the child to get to the nearest referral centres ranges from one to 15 h, and access to transport is very poor. This is a huge challenge for a country like Ethiopia, which strives to ensure universal health coverage [17]. Survey results show that drugs should be available at first-level health facilities when referral is impossible [29].

Timely forecasting and quantification is a key factor for the distribution of drugs and supplies [17], but health extension workers do not send essential drug supply requests to higher levels in Ethiopia [30]. In 23 countries, including Ethiopia, many health workers in health posts referred severe pneumonia cases without administering pre-referral drugs [31]. One possible reason for this might be the lack of these drugs in the facilities. Moreover, only 60% of the health workers refer the case scenario with pre-referral drugs in our study.

Very few health facilities had all the job aids and supplies needed for the management of pneumonia. In previous study from Ethiopia, supplies of commodities in health posts have been found to be weak [32]. As compared to a study conducted in three sub-Sahara Africa countries, the availability of thermometers, syringes and needles at health centres are similar to our findings [29]. In Ethiopia, out of 91% of health posts that had a functional timer, 100% had an IMNCI chart booklet and 80% had a thermometer [26], which is similar to our findings, with the exception of the functional timer, in which only 24% of the health posts had this material. A functional timer is a crucial device for correctly counting respiratory rate, and accurately identifying children with fast breathing for the management of pneumonia. In addition, IMNCI job aids have an impact in improving the performance of health professionals in providing quality child care [11]. From our findings, 3% of health centres had pulse oximetry and 9% had an oxygen cylinder/concentrator. Findings are from a systematic review concluded that health facilities are lacking the basic equipment for an effective management of hypoxemia in childhood pneumonia [33].

The proportion of staff trained in IMNCI and managing children at health centres on the day of visits was only 11%. As a standard, the Ethiopian government planned to have at least two health workers trained in IMNCI in each health centres [17], but our finding showed that on average there are only 1.9 health workers trained and deployed per health centres. One of the main programmatic IMNCI indicators to measure the implementation of training is a 60% threshold of trained personnel in a given first-level health facility [23], but only 60% of the health centres had achieved this threshold. The proportion of health extension workers trained in managing children was far beyond the global standard, as there should be six community health workers trained in iCCM and deployed in health posts per 1000 children under 5 years of age [24]. But our finding showed that there are approximately 1.2 health extension workers per 1000 children less than 5 years of age.

The low availability of vaccines and supplies found in health posts is a major bottleneck for the expanded immunization programme in Ethiopia. Our finding shows that only 18% of the surveyed health posts had supplies to support vaccines services. But all the surveyed health centres had these supplies, which is higher than findings from three other Sub-Saharan Africa countries [29]. In Ethiopia, the provision of vaccines is challenged by a shortage of supplies, vaccines stock outs and cold chain breakages [17]. In addition, vaccines services were not available for the entire week in the study area.

The low proportion of health extension workers who correctly classified the case scenario according to the IMNCI algorithm is comparable with the study in Uganda, in which 75% of community health workers correctly classified pneumonia cases [13]. Because of the methodological approaches being used in assessing the performance of health workers to classify severe pneumonia, it is very difficult to compare our findings. However, the low proportion of health workers who correctly classified the case scenario is supported by findings from South Africa. In this study, which used the direct observation of consultations and a re-examination of cases, only 48% of children were correctly classified as having severe pneumonia by health workers [34]. Few health workers identified the correct treatment for the case scenario, which is supported by other findings that used records, direct observation and the re-examination of cases [35, 36].

Number of supervision visit with direct observation of case management has impact on providers’ knowledge on management of severe pneumonia. This is supported by a finding from trial study that support like supervision for providers ensures better child health care [11]. In Ethiopia most of the health workers at health posts are women who have one year theoretical and practical training [18]. In our survey majority of the health workers (89%) are women from health posts. The large proportion of women health workers with one year duration of training may be one possible reason for the association between gender and knowledge in management of severe childhood pneumonia.

## Conclusions

The findings of this survey show that the current state of health system support for managing childhood pneumonia in the context of IMNCI/iCCM in Ethiopia is weak. The Federal Ministry of Health aims to reduce the child mortality rate to 30 per 1000 live births by the year 2020 [17]. To help achieve this goal, the government has promised to increase the availability of essential drugs to 100% at all levels of facilities, and to ensure the availability of an uninterrupted commodity supply. Results from our survey imply that much remains to be done, at least in the study area. So the findings of this study will help the effort already made by the government to ensure supply chain management of drugs, vaccines, and equipment, and Strengthen supportive supervision, at the health facility. Nonetheless, the knowledge of healthcare providers in managing severe pneumonia still needs to be improved through training and a research that pragmatically test interventions that could improve the management of childhood pneumonia.

### Unanswered questions and future research area

The indicators that we included in our study are inputs that are keys to providing quality child care for the management of pneumonia. The level of quality service provided by the health workers for children with pneumonia is still an unanswered question. Therefore, assessing the capacity of health workers in managing children with pneumonia through a direct observation of consultations and a re-examination of cases by clinician is a future research area. It is not known how long the surveyed health facilities were out of stock for essential drugs, equipment and supplies. For this reason, research on the number of stock out days, and reasons for stock out might be needed in the future. Since our survey didn’t assess the possible challenges the health workers encountered, we recommend future research to focus on challenges health care providers faced.

## Data Availability

The datasets used and/or analyzed during the current study are available from the corresponding author on reasonable request.

## Abbreviations

BCG: Bacille Calmette Guerin
CHW: Community Health Worker
DPT: Diphtheria, Pertussis, and Tetanus
HepB: Hepatitis B
HEW: Health Extension Worker
iCCM: integrated Community Case Management
IMCI: Integrated Management of Childhood Illness
IMNCI: Integrated Management of Neonatal and Childhood Illness
PCV: Pneumococcal Conjugate Vaccine
SPSS: Statistical Package for Social Sciences
UNICEF: United Nations International Children’s Emergency Fund
WHO: World Health Organization
